# Naturally occurring influenza reassortment in pigs facilitates the emergence of intrahost virus subpopulations with distinct genotypes and replicative fitness

**DOI:** 10.1128/mbio.01924-24

**Published:** 2024-11-29

**Authors:** Chong Li, Victoria Meliopoulos, Aaron Rendahl, Stacey Schultz-Cherry, Montserrat Torremorell

**Affiliations:** 1 College of Veterinary Medicine, University of Minnesota, St. Paul, Minnesota, USA; 2 Department of Host-Microbe Interactions, St. Jude Children’s Research Hospital, Memphis, Tennessee, USA; McMaster University, Hamilton, Ontario, Canada

**Keywords:** influenza A virus, reassortment, replicative fitness, pig, swine farm

## Abstract

Pigs are recognized as amplifying hosts for influenza A virus (IAV) reassortant viruses. Understanding the extent of IAV reassortment occurring at the individual pig level in naturally infected pigs and how reassortment impacts virus diversity, persistence, and replicative fitness is essential for countering IAV threats to humans and animals. Here, 244 IAV plaques were isolated from 24 commercial pigs, resulting in 26 distinct genome segment constellations. About 33% (8/24) of pigs were infected with two or more different genotypes, with two pigs harboring two or more different subtypes. Our results indicate that few pigs in a population harbor significantly more genotypes than other pigs and generate most of the diversity, including the emergence of reassortants. However, detecting distinct genotypes during surveillance was dynamic, with most of the genotypes subsiding over time. All the IAV genotypes could replicate in various swine and human-sourced respiratory epithelial cells, and we observed that distinct reassortant genotypes recovered from a single pig could exhibit different growth abilities, especially in human cells. Overall, we demonstrated that multiple distinct IAV genotypes with distinct antigenic profiles and varying growth abilities on swine and human respiratory tracts can be shed simultaneously from a single pig, which contributes to the dynamic nature of IAV prevalence. The striking magnitude of IAV reassortment at the single pig level revealed in this study highlights the need to strengthen surveillance efforts and plans to eliminate IAV from swine farms because pigs have a high potential to produce diverse and potentially zoonotic influenza reassortant viruses.

**IMPORTANCE:**

Pigs play a crucial role in driving influenza A virus (IAV) diversification and evolution by reassorting the viruses originating from different hosts. Despite IAV reassortment and diversity being well documented in pig populations at different scales (e.g., farm, region, country), limited field research has explored the extent of reassortment happening at the single pig level and how that contributes to the overall genetic and biological variation observed in populations. We provide initial information on levels of reassortment happening at the single pig level in naturally infected pigs, and that particular pigs can shed a plethora of distinct genotypes, with certain genotypes having distinct replicative fitness on swine and human respiratory tracts, which preserves the potential for IAV long-term evolution and facilitates the emergence of zoonotic/pandemic-capable reassortants.

## INTRODUCTION

Influenza A virus (IAV) reassortment is a major driver for virus diversification that facilitates the emergence of novel viruses that cause zoonotic infections and even influenza pandemics (1–3). Differing from genetic drift that gradually accumulates point mutations on the virus genome during replication, reassortment is more efficient at modifying the virus’s genetic components and expedites virus evolution by swapping intact gene segments between multiple viruses during co-infection (4, 5). By integrating the IAV gene segments from antigenically distinct viruses or from influenza viruses adapted to different hosts, reassortment has played a prominent role in producing novel reassortants that cause immune evasion and cross-species infection, including the most recent pandemic in 2009 (6–8). Although reassortment is constrained by the incompatibility between virus components that derive from parental viruses, IAV reassortment happens frequently in nature on multiple host species, making control of influenza difficult for both animal and public health officials (5, 9).

Both, pigs themselves and the rearing conditions of the pigs, facilitate the emergence of novel reassortants (10). Pigs are susceptible to IAVs from multiple host species and can serve as mixing vessels for human, avian, and swine-origin IAVs that can reassort with each other (11, 12). Several experimental studies have shown that single pigs can generate a significant amount of IAV reassortants relatively quickly after infection under different challenge models (13–15). In addition to the pig itself, the complex ecosystem and herd management practices taking place in swine, position swine herds as ideal locations for maintaining influenza infections endemic, facilitating the emergence of new strains (16–18). The dynamic nature of swine herds, including the commingling of pigs of various ages with varying immunity and infection statuses, and pig movement within or between various swine holdings, facilitates the continual introduction and transmission of multiple distinct IAVs among susceptible animals, which provides an optimal ground for virus reassortment to occur in the pigs (16, 19). As a result, co-circulation of distinct IAV subtypes in pig farms is common, and significant genotypic and antigenic diversity of IAV exists from farm to farm (17, 19–21). Over 74 distinct genotypes have been documented for H1 virus in US pigs alone during 17 years of surveillance (22). The extensive genotypic diversity has also been observed in H3 IAVs, particularly from the various combinations of the “TRIG” (triple-reassortant internal gene) cassette and the multiple hemagglutinin and neuraminidase lineages (23, 24). Besides, 17 IAV genotypes circulated in Danish swine herds from 2011 to 2018, with multiple genotypes harboring IAV segments from H3N2 human seasonal and various swine-origin IAV viruses (25). Most of our understanding of the presence of IAV reassortants in pigs has been acquired through passive surveillance efforts or disease investigations at the farm, region, or national level or learned from studies on individual pigs under experimental conditions. However, the extent of reassortment events occurring at the single pig level in farms and how it affects the diversity of IAV subpopulations harbored in single pigs is largely unknown.

Tissue tropism has been speculated to be a crucial determinant for the emergence and distribution of IAV reassortants along the respiratory tract and affects the viruses’ efficiency of onward transmission among hosts (14, 15, 26). Generally, IAV that replicates in the upper respiratory tract represents the virus population that causes the initial infection and transmission in mammal hosts, while the IAV that propagates in the lower respiratory tract may lead to severe disease outcomes (15, 26–28). The growth performance of IAVs is greatly affected by the variability of temperature and presence of multiple types of IAV receptors between different parts of the respiratory tract, and the difference in replicative fitness between IAVs contributes to respiratory tissue tropism (15, 29). Several studies have revealed the impact of tissue-specific differences on the probability of IAV reassortment within different animal models. In ferrets, higher diversity was detected in IAV reassortant populations obtained from nasal turbinates compared to lung tissues (14). Evidence of association between tissue tropism and IAV reassortment has also been documented in pigs, as one study reported more reassortants generated in the middle and lower respiratory tract compared to the relatively few found in upper respiratory tissues (15). Besides, the swine nasal cavity appears to play a key role in selecting reassortants that can be transmitted onward because only a small portion of reassortants could replicate in swine nasal epithelial cells at 33°C (15). Furthermore, naturally occurring swine IAVs have been documented to have diverse tissue tropisms for the human respiratory tract, and genotype changes occurring through reassortment could significantly alter or expand their tissue tropism for human upper airways (30). Therefore, it is important to understand the tissue tropism of swine IAVs in swine and human respiratory tracts to assess their risk of onward transmission among pigs and people, which begs for more studies designed to test the growth characteristics of reassortants obtained from naturally infected pigs under farm settings.

This study aims to identify IAV reassortants happening at the single pig level under field conditions and evaluate their genotypic diversity and replicative fitness to better understand the emergence, persistence, and subsidence of influenza viruses in domestic pig populations and risk of zoonotic infections. We used nasal swabs obtained from pigs in a commercial swine farm with documented extensive subtype IAV co-circulation during a 6-month surveillance (17). Here, we performed viral plaque assays on nasal swabs collected from single pigs, purified IAV subpopulations from each pig, categorized IAV genotypes with multiple gene constellations, and tested the replicative fitness of distinct IAV genotypes in various cell lines sourced from upper, middle, and lower parts of swine and human respiratory tracts grown as monolayers and air–liquid interface conditions.

## RESULTS

### Background information of field samples

The nasal swabs used for this study originated from a published research that compared the genetic variability of circulating IAVs among 23 breed-to-wean swine herds by directly sequencing the nasal swabs collected from the piglets before weaning (around 18 days old) (17). The staff at each farm identified one pig per litter and collected the nasal swabs for a total of 30 nasal swabs collected monthly. Up to 180 nasal swabs were collected from each herd. For the purposes of our study, we selected one farm that had extensive evidence of multiple subtype IAV co-circulation during the 6-month surveillance period. In addition, the sequences obtained from the selected farm exhibited high level of nucleotide polymorphism and genotypic diversity (17). We used all the 48 nasal swabs that had tested RT-PCR IAV positive and from which we had obtained whole-genome sequences, and used those samples to identify virus subpopulations in single pigs through plaque assay.

### Multiple genotypes identified among viral plaques purified from single farm pigs

**Fig 1:**
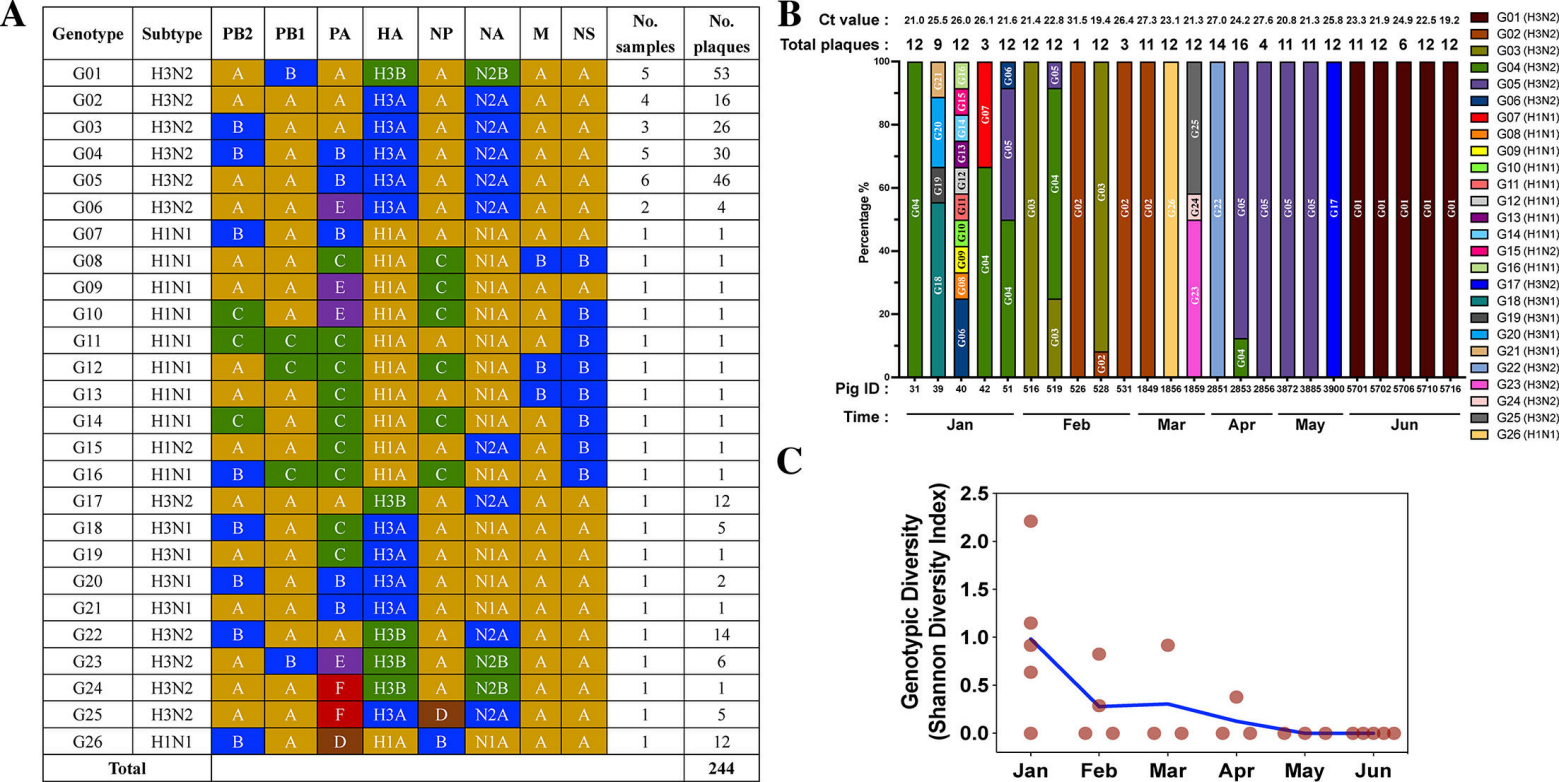
Summary of genotypes of the plaque-purified influenza A viruses. (**A**) A total of 244 influenza plaques were whole-genome sequenced and genotyped based on the clade assignment of each gene segment. Each row represents specific virus gene constellation, and the different clades within each gene segment are displayed in different colors with clade IDs (A, B, C, etc.) as determined by PhyloPart. Color and ID for each clade of the different influenza gene segments are defined in Figure S1. No evolutionary relationship is inferred if the clades of distinct gene segments are assigned the same colors and IDs. The quantity of influenza plaques for each genotype and the number of samples that these plaques were isolated from are indicated on the right side of each row. The G-number (genotype number) on the left column indicates a specific genotype number for the influenza plaques. (**B**) Genotype distribution of influenza A plaque viruses obtained from each individual pig. Each bar represents the percentage of different genotypes detected (Y axis) by pig ID number (X axis). The colors indicate different genotypes of the viral plaques. The collection time of each sample (in months) is indicated below the corresponding pig ID. The Ct value of the nasal swab (pig) collected from the corresponding pig used for the plaque assay was measured by real-time RT-PCR targeting the influenza matrix gene and indicated above the bar. (**C**) Genotype diversity measured by the Shannon-Weiner index for influenza plaques isolated from each pig throughout the surveillance period. The Shannon-Weiner diversity was calculated for each pig (except for pig #526 because only one influenza plaque was isolated from the sample) and is displayed by individual dots based on the quantity and frequency of each influenza genotype identified in each pig. The viral genotypic diversity is displayed by month when the samples were collected, and the blue line connects the average diversity calculated among samples collected in each corresponding month.

Among the 48 IAV RT-PCR positive nasal swabs collected from the farm, 24 of them could be plaque purified, yielding a total of 244 IAV plaque isolates. These isolates included 213 H3N2, 9 H3N1, 21 H1N1, and 1 H1N2 viruses (H3 – C IVA, 1990.4.a lineage; H1 – gamma-c3, 1A.3.3.3-c3 lineage; N2 – 2002A lineage; N1 – classicalSwine, N1.C.3 lineage). Based on the clade assignment criteria (see Materials and Methods), we identified 26 genetic clades from the phylogenetic trees for the eight IAV gene segments of the plaque isolates (Fig. S1), which categorized the 244 IAV plaques into 26 distinct genotypes (Fig. 1A). Most of the genotypes (~77%, 20/26) were only detected in single pigs, and about 23% (6/26) of genotypes could be found in multiple pigs representing 72% (175/244) of the isolated IAV plaques (Fig. 1A).

### Virus subpopulations shed from single pigs exhibited high genotypic and antigenic diversity

To understand the extent of potential reassortment events that could happen in single pigs, we compared and quantified the genotypic diversity of IAV plaques isolated from each pig. About 33% (8/24) of pigs had IAV plaques with two or more distinct genotypes, and out of these, two pigs had IAV plaques with two or more distinct subtypes (sample from pig #40 had 8 H1N1, 1 H1N2, and 3 H3N2 virus plaques; sample from pig #42 had 1 H1N1 and 2 H3N2 virus plaques) (Fig. 1B). About 75% (6/8) of pigs with multiple IAV genotypes were from the first 2 months of the 6-month virus surveillance. We measured the IAV genotypic diversity at the single pig level by calculating the Shannon diversity index, which measures how diverse a sample is. We found that the genotypic diversity of IAV detected in single pigs continued to decrease during the surveillance period (Fig. 1C). Although it was based on a limited number of pigs, we observed a rapid and extensive genotypic virus shift in individual pigs between months. In addition, the results on the dynamics of IAV genotypic diversity at the single pig level during the surveillance period are concordant with our previous research, which explored the change of IAV diversity at the population level based on the direct sequencing on the same set of nasal swabs (17).

**Fig 2:**
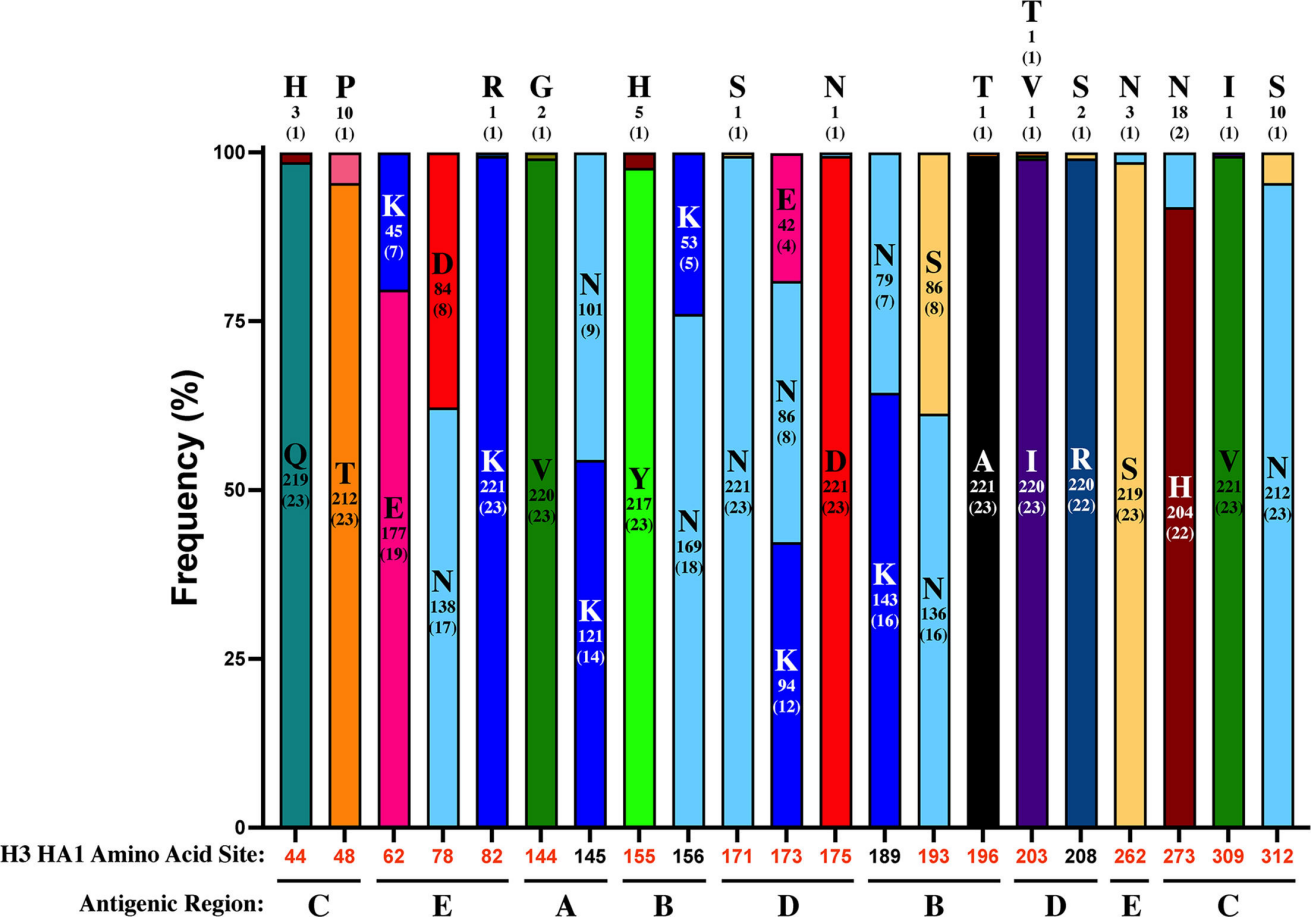
Plaque-purified swine influenza A viruses exhibit various amino acid profiles on H3 antigenic sites. Twenty-one H3 antigenic sites presented polymorphic amino acids among 222 H3 subtype influenza plaques isolated from 23 pigs. The frequency of each amino acid on certain polymorphic antigenic sites (HA1 numbering) among H3 influenza plaques is displayed using stacked bar plots. The number under each amino acid abbreviation and the number in parenthesis indicates the number of plaques and the number of samples from which plaques were isolated containing the corresponding amino acid on the specific antigenic site, respectively. The red amino acid site indicates that the corresponding amino acid polymorphism is presented in the plaques isolated from the same sample. The H3 antigenic regions are identified below the amino acid sites based on reference (31). We did not detect any amino acid polymorphism on the H1 antigenic regions among the remaining 22 H1 subtype plaque isolates from the three pigs analyzed.

To explore the antigenic diversity of IAV shed from pigs, we compared the amino acids on the hemagglutinin (HA) antigenic sites among the isolated plaques. All the 22 H1 subtype plaque isolates from three pigs displayed the same amino acid profile on the H1 antigenic regions. In contrast, the 222 H3 subtype IAV plaques from 23 pigs exhibited amino acid polymorphisms on 21 amino acid sites in H3 antigenic regions (Fig. 2). Additionally, 17 H3 antigenic sites had distinct amino acids among IAV plaques isolated from the same pig (Fig. 2), and about 52% (12/23) of pigs were shedding H3 subtype viruses with multiple distinct amino acid profiles on the H3 antigenic sites.

### Differences in replicative fitness of distinct genotypes of influenza observed in human and swine respiratory cell monolayers

To test the replicative fitness and whether the different genetic combinations of naturally occurring swine IAVs could affect their tissue tropisms in swine and human respiratory tracts, we selected different cell lines to represent the upper, middle, and lower human and swine respiratory tracts. Epithelial cells sourced from swine lungs were not available and could not be included in this study. We measured the growth kinetics of 21 representative IAV plaque isolates from 21 distinct genotypes on human nasal primary epithelial cells (HNEpC) and swine nasal primary epithelial cells (sNEC) at 33°C; on human bronchial/tracheal epithelial cells (NHBE) and normal swine bronchial epithelial cells (NSBE) at 37°C; and on human lung carcinoma epithelial cells (A549) at 39°C.

**Fig 3:**
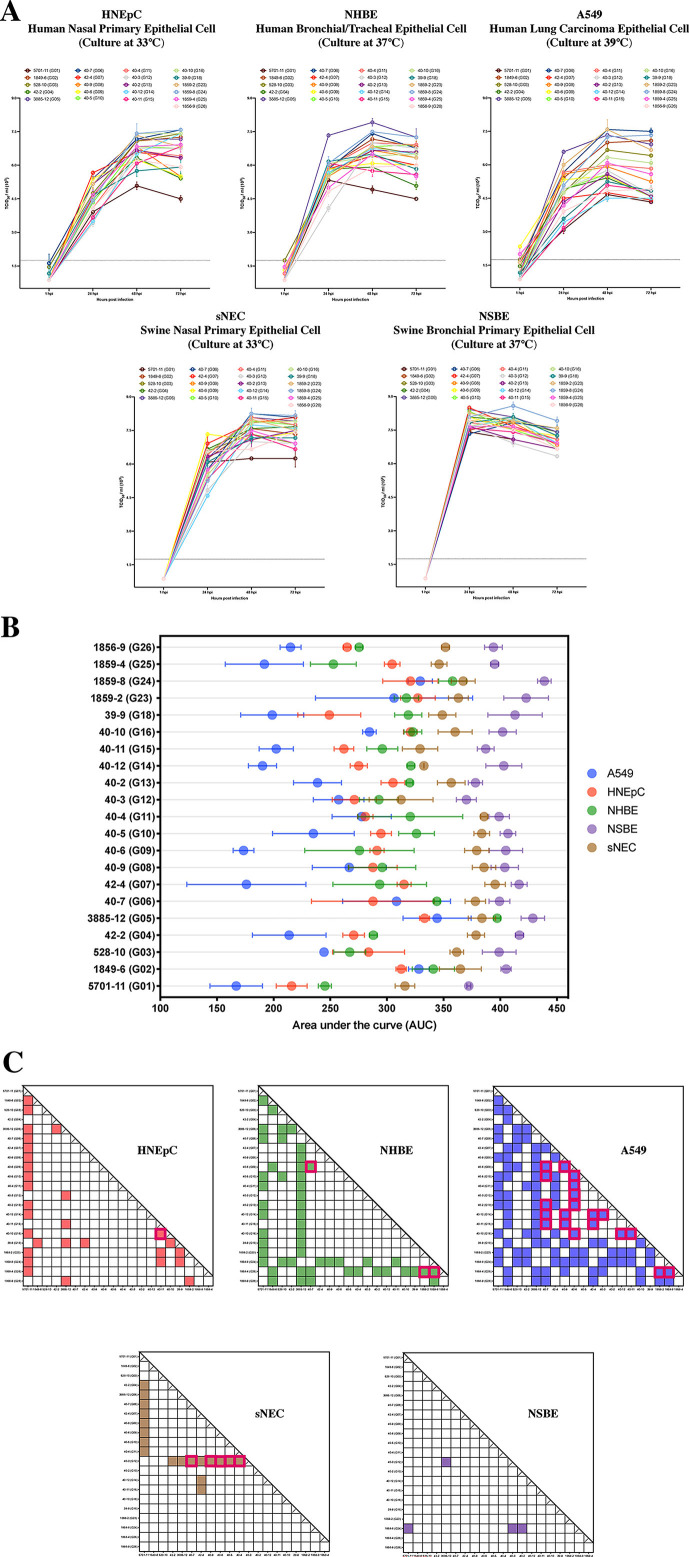
Growth performance of influenza A viral plaques with distinct genotypes on various human and swine respiratory epithelial cell monolayers. (**A**) Influenza growth kinetics of plaque-purified isolates on the normal swine bronchial epithelial cell (NSBE), swine nasal primary epithelial cell (sNEC), human bronchial/tracheal epithelial cell (NHBE), human nasal primary epithelial cell (HNEpC), and human lung carcinoma epithelial cell (A549) grown as monolayers. The cells were infected by indicated viruses at 33°C, 37°C, or 39°C with a multiplicity of infection of 0.01 TCID_50_/cell. The supernatants of infected cells were collected at 1, 24, 48, and 72 hours postinoculation (hpi), and the virus titers were determined by TCID_50_ assay. The tested influenza viruses are shown in different colors, and the data are shown as mean titers ± standard errors of three replicates. (**B**) The 21 influenza viral plaques representing 21 genotypes were inoculated on NSBE, sNEC, NHBE, HNEpC, and A549 cells at a multiplicity of infection of 0.01 TCID_50_/cell and were cultured at either 33°C, 37°C, or 39°C. We computed the area under the curve (AUC) for each virus based on the virus growth kinetic curves shown in Figure 3A. The AUC values of each viral plaque are displayed as the mean values ± standard deviations (*n* = 3 replicates). Each cell line is represented by a different color. (**C**) Statistical differences compared the average AUC values among all the 21 influenza genotypes for each cell line using two-way ANOVA with Tukey HSD for multiple group comparisons. The X and Y axes indicate the ID of the plaques with (Y label) or without (X label) the genotypes they represented from that used for each pairwise comparison. The colored squares indicate statistical differences (*P* < 0.05) of the average AUC values between two viruses. The colored squares with pink borders indicate the significant differences in AUC values of two viral plaques/genotypes isolated from the same pig.

The selected isolates of all genotypes could replicate in various human and swine respiratory cell monolayers (Fig. 3A). To compare the replicative fitness between different IAV plaques on various cell lines, we calculated the area under the curve (AUC) based on the 50% tissue culture infectious dose (TCID_50_) values for each IAV plaque isolate and cell monolayer at various times of growth. The plaque viruses generally showed higher replicative kinetics in swine respiratory cells (NSBE and sNEC) compared to human respiratory cells (HNEpC, NHBE, and A549), and we observed more variability in IAV viral titers among isolates in human-sourced respiratory cells than in swine-sourced epithelial cells (Fig. 3B and C). Among the human cell lines, most IAV isolates grew better in primary epithelial cells sourced from nasal cavities (HNEpC) and bronchi (NHBE) than cells from human lungs (A549). Furthermore, the IAV isolates exhibited more variation in the replicative fitness on the cell monolayers from the lower than the upper human respiratory tract (Fig. 3C). In addition, we observed that the distinct genotypes of IAV shed from pig #40 or pig #1859 exhibited different growth abilities on HNEpC, NHBE, A549, and sNEC cells grown under monolayer conditions.

### Influenza viruses with different gene combinations displayed efficient replication in swine and human respiratory cells at the air–liquid interface

The air–liquid interface (ALI) culture exposes the respiratory primary epithelial cells directly to the air, which provides an alternative model for influenza research to *in vivo* or traditional *in vitro* cell culture experimentations. In general, ALI cultures better simulate the processes of cells reacting to influenza infections in the atmosphere of the host airway (32). In this study, we characterized the growth properties of 23 IAV plaque viruses from 21 genotypes using both a human bronchial/tracheal epithelial cell (NHBE-ALI) and a swine tracheal primary epithelial cell (sTEC-ALI) grown under air–liquid interface conditions.

**Fig 4:**
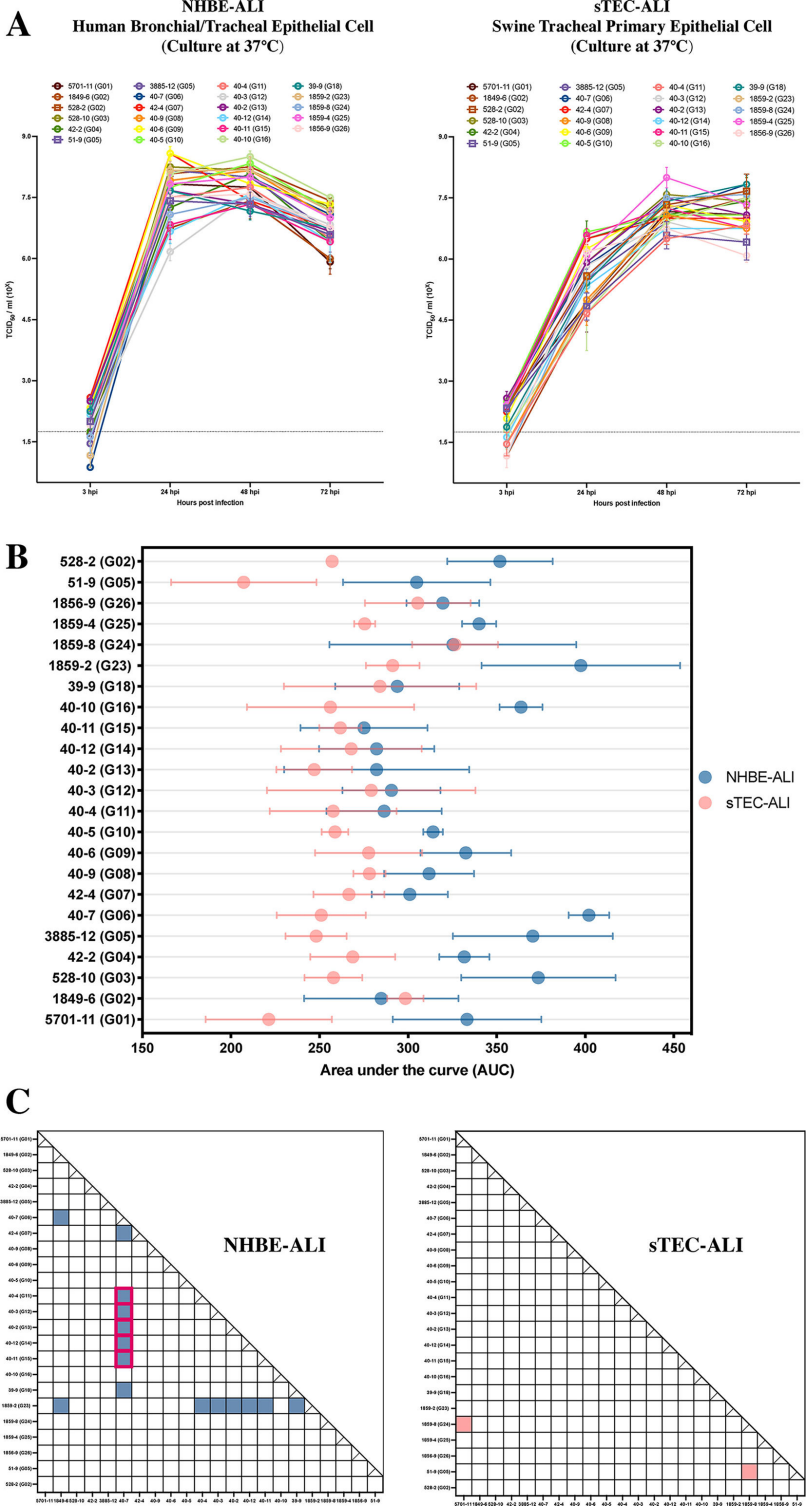
Growth performance of influenza plaques with distinct genotypes tested on swine and human respiratory primary epithelial cells at the air–liquid interface. (**A**) Influenza growth kinetics of selected plaque-purified isolates on human bronchial/tracheal epithelial (NHBE-ALI) and swine tracheal primary epithelial (sTEC-ALI) cells at the air–liquid interface. The selected representative influenza viruses were inoculated on the cells at 37°C with a multiplicity of infection of 0.1 TCID_50_/cell. The apical supernatants were obtained at 3, 24, 48, and 72 hours postinoculation (hpi), and the viral titers were determined with TCID_50_ assay. The influenza viruses are displayed in different colors and shapes. Error bars present the standard error of the means from three replicates. (**B**) The NHBE-ALI and the sTEC-ALI cells were cultured and inoculated in 37°C under air–liquid interface conditions with 23 influenza plaque viruses representing 21 genotypes at a multiplicity of infection of 0.1 TCID_50_/cell. The area under the curve (AUC) was calculated based on the virus growth kinetic curves shown in Figure 4A. (**C**) Statistical analysis on the average AUC values among the 23 influenza plaques tested on each cell line using two-way ANOVA with Tukey HSD adjustments. The figure settings of panels A, B, and C are the same as the corresponding figure panels in Figure 3.

In general, although the selected IAV plaques generally had higher replicative kinetics on NHBE-ALI than in sTEC-ALI cells (Fig. 4A and B), all the IAV plaques representing 21 genotypes showed high growth abilities in NHBE-ALI and sTEC-ALI cells at the air–liquid interface, with the peak average virus titer of 7.79 (±standard deviation; ±0.52, 48 hours postinoculation [hpi], NHBE-ALI) and 7.15 (±0.62, 72 hpi, sTEC-ALI) log_10_ TCID_50_/mL, respectively (Fig. 4A). Consistent with the results of virus growth obtained in cell monolayers, we observed relatively large variations in replicative kinetics among IAV isolates grown on NHBE-ALI compared to sTEC-ALI cells. However, the overall variation in growth titers among viruses tested in swine and human respiratory cells at the air–liquid interface was lower than those tested in cell monolayers. Furthermore, plaques with distinct genotypes isolated from pig #40 exhibited distinct replicative fitness on NHBE-ALI cells (Fig. 4C).

## DISCUSSION

The wide susceptibility of pigs to IAVs from multiple hosts positions the pig as a central intermediate host capable to expand viral diversity and drive virus evolution by reassorting the genetic components from different influenza viruses. The constant changing IAVs in pig populations significantly elevate the difficulty to prevent and control influenza infections in pigs and represent a persistent proximal source of zoonotic threats to public health. Surveillance of IAVs in pigs together with laboratory-based research on the pathogenesis and infectivity of newly emerged viruses is urgently needed to track the evolutionary dynamics and diversity of IAV populations *in situ* and help with effective pandemic preparedness (33). However, limited studies have revealed the level of IAV reassortment that contributes to the expansion and diversity of virus genotypes found in single pigs housed under farm conditions and the degree of variability in the infectivity and replicative fitness of these genotypes on swine and human respiratory cells. To fill these knowledge gaps, we collected nasal swabs from pigs in a farm during a 6-month surveillance and plaque purified 244 IAV isolates recovered from 24 pigs that grouped into 26 distinct genotypes. The viral plaques exhibited distinct H3 antigenic profiles on 21 polymorphic antigenic sites. Among them, there were six sites (HA1 sites 62, 144, 145, 156, 189, and 193) that could bind to mouse monoclonal antibodies, and four codons (HA1 sites 145, 156, 193, and 262) were identified as being positively selected in previous studies (31, 34–36). In addition, we documented that one-third of the tested pigs shed multiple distinct genotypes of IAVs simultaneously. All the IAV plaques recovered from pigs could replicate on various swine and human respiratory cells under monolayer and air–liquid interface conditions, indicating their zoonotic potential. However, the distinct influenza genotypes displayed lower but more variable viral titers on monolayers of human respiratory epithelial cells compared to swine respiratory cells. Our study addresses the need to pursue efforts to strengthen IAV surveillance and restrain the IAV genetic diversity in swine, as single pigs can harbor IAV subpopulations with distinct genotypes, antigenic profiles, and replicative fitness in swine and human respiratory tracts, which represents a threat to swine production and public health.

Virus surveillance in the swine industry is essential for effective disease control in pig herds and also critical for human public health because pigs harbor multiple zoonotic pathogens capable of causing outbreaks in human populations (10). Among these pathogens, the zoonotic threat from swine influenza viruses is especially considerable due to the frequent bidirectional transmission and tangled evolutionary relationship of IAV between swine and humans (37). Reports of human IAV infections linked to pigs and the latest IAV pandemic events led by the 2009 pH1N1 virus reminded us of the role that pigs play at generating novel reassortant viruses with zoonotic and pandemic potential (1, 38–42). Meanwhile, the recurrent introduction of human IAVs into pigs further overturn the topography of circulating viruses and makes the evolution of IAV in pigs unpredictable (43, 44). Influenza infections in human and animal populations are hard to prevent due to the virus’s exceptional ability to change (45). The plasticity of the segmented genome enables the IAV population to maintain a high level of genetic variation by producing IAV viruses with different genotypes and mutational profiles, which confers a benefit to the IAV populations in dealing with dynamic or adverse environments. As the primary genetic mechanism for IAV evolution, reassortment can change the virus genome and biological properties remarkably in a relatively short time and contribute significantly to the virus’s genetic diversity. In swine breeding herds, the gilts (young adult female pigs for herd replacement) and the pre-weaned piglets are considered major pig subpopulations to introduce infections of new IAV strains and facilitate the co-circulation of IAVs with distinct genotypes at the farm level (18). However, the magnitude and extent of IAV reassortment in pigs evaluated by farm-level field research is likely underestimated because it mainly captures the most prevalent or easily cultivated reassortants. The major circulating reassortants usually exhibit superior functionality under normal conditions, while the minor genotypes may present the advantage of survival and adaptation in certain circumstances like vaccination or drug treatment (45). Therefore, the frequency of reassortment events in swine farms needs to be explored at the single pig level to fully grasp the degree of reassortment happening in pigs. Even though the IAV subpopulations have already gone through bottleneck processes selected most likely through replication in the swine respiratory tract and shedding into the environment (15), our study provides the initial evidence of potential reassortment in farm settings that result in multiple IAV genotypes being shed at the single pig level. Despite the variability in replicative kinetics, all the representative plaque isolates with distinct genotypes could replicate in cells sourced from various locations in swine and human respiratory tracts. We found that about 33% of pigs were harboring multiple distinct genotypes of IAVs. Although these pigs did not contribute the same to the generation of different genotypes of reassorted viruses, there were some “super-reassortant” pigs, like pig #40 that harbored more genotypes than other pigs, which is consistent with the findings in a previous study that explored the IAV reassortment in pigs under experimental conditions (13). These observations are especially noteworthy considering that the US pig inventory exceeds 70 million head, and distinct IAVs co-circulate in US pig farms that could reassort with each other (17, 18, 46). Swine populations have an enormous potential to facilitate the emergence of new IAV strains with zoonotic threats and contribute significantly to long-term viral evolution.

Testing the replicative fitness of IAV reassortants is an essential first step to assess whether novel reassortants can survive in the environment and transmit onward among host populations. The fitness of IAV reassortants with specific genotypes depends on the compatibility of functional proteins and package signals from gene segments (5). IAV reassortment has a fitness cost, and usually newly reassorted viruses are less fit than parental viruses. Still, the progeny of reassortants must undergo selection by adapting and competing with the parental viruses within the same environment (26). As a result, the IAV reassortants will encounter mild negative selection and are more likely to be outgrown in the environment if they have minimal genetic distance from their parental viruses in the steady environment (47). On the other hand, IAVs with remarkable genome change through reassortment may quickly dominate if the environmental conditions are undergoing significant changes, like vaccination, drug treatment, and spillover infection. In this study, we observed that the patterns of IAV genotypes present in single pigs shifted over time, and these swine-origin IAVs generally grow better in the swine respiratory tract than in the human respiratory tract. Additionally, the difference in IAV gene constellations seems to affect virus replication in the human respiratory tract because the tested IAVs with distinct genotypes exhibited more variations in growth ability in human cells compared to swine cells. It is noteworthy that genotype 1 (G01) H3N2 virus had not been detected in pigs during the first 5 months of the study, but then it became the only predominant genotype circulating in the farm during the last month of surveillance. However, compared to viruses from the other genotypes, we did not observe a superior replicative fitness of the representative G01 strain on human and swine-origin respiratory cells. On the contrary, the selected G01 viruses exhibited one of the lowest replicative kinetics among the viral plaques we tested on the human-sourced primary respiratory cells. The sudden appearance and dominance of the G01 genotype may be due to its difference in antigenic properties as the amino acids of the particular G01 tested isolate differ from selected plaques of other genotypes on multiple H3 antigenic sites (H3 HA1 sites 62, 78, 145, 156, 173, 189, and 193). Considering these sites (except for sites 78 and 173) have been recognized as the mouse monoclonal antibody binding and/or positively selected codons in previous studies (31, 34–36), it is likely that these immunodominant positions determine the virus antigenic properties and contribute to the virus prevalence. However, other epidemiological and biological factors may have affected its prevalence in pigs, such as farm management practices, IAV receptor-binding properties, IAV acid stability, IAV transmissibility among pigs, and the history of IAV natural infections in the pigs. Additionally, although the genomes of the tested strains were highly homologous to the consensus genome of their genotypes, genetic variations existed among the virus plaques within the same genotypes. Further studies that test a larger number of isolates of the same genotype *in vivo* and *in vitro* should help validate the variations of biological properties between viruses with different genotypes and better evaluate their potential threat to animals and public health.

Understanding the genotypic diversity and biological characteristics of IAV shed from single pigs is essential to unravel how the existence of multiple virus subtypes and genotypes is maintained at the population level. The results of our study highlight the need for IAV surveillance and measures to limit the virus genetic diversity in pigs to counter the virological threats to the swine industry and public health. Although we cannot identify the progeny reassortants and parental viruses from the purified plaque isolates in the single pigs due to lack of information on reference background strains under field conditions, we showed new evidence that pigs have a high potential to generate diverse IAV viruses through reassortment by shedding IAVs of various genotypes and replicative fitness simultaneously. Further research that includes a larger number of pigs and conducting surveillance through tracing the pig flows across multiple production stages are needed to provide advanced knowledge on the expansion of IAV genotype diversity and the factors that drive the emergence of IAV reassortants at the single pig level under farm conditions.

## MATERIALS AND METHODS

### Cell lines

The Madin-Darby canine kidney (MDCK) cell line was obtained from the University of Minnesota Veterinary Diagnostic Laboratory (VDL) and was originally purchased from the American Type Culture Collection (ATCC, CCL-34). MDCK cells were maintained in Dulbecco’s modified Eagle medium (DMEM, Gibco, Carlsbad, CA, USA) supplemented with 10% fetal bovine serum (FBS, Gibco, Carlsbad, CA, USA), 1% streptomycin-penicillin (Gibco, Carlsbad, CA, USA), 1% HEPES (Gibco, Carlsbad, CA, USA), and 1% sodium pyruvate (Gibco, Carlsbad, CA, USA). The human lung carcinoma epithelial cell line (A549, CCL-185) was purchased from ATCC and cultured in DMEM media with 10% FBS and 1% streptomycin-penicillin. The HNEpC (C-12620) was purchased from PromoCell (Heidelberg, Germany), and cells were grown in airway epithelial cell growth medium (C-21060, PromoCell). The NHBE with retinoic acid (CC-2540) and NHBE cells for B-ALI Culture (NHBE-ALI, CC-2540S) were purchased from Lonza and maintained in BEGM bronchial epithelial cell growth medium (CC-3170, BEGM Bronchial Epithelial Cell Growth Medium BulletKit, Lonza) and B-ALI growth medium (00193514, B-ALI Bronchial Air-Liquid Interface Medium BulletKit, Lonza), respectively. The swine primary respiratory epithelial cells, including sNEC, swine tracheal primary epithelial cells (sTEC-ALI), and NSBE, were kindly provided by Dr. Stacy Schultz-Cherry from St. Jude Children’s Research Hospital, Memphis, TN, USA. All the swine primary respiratory epithelial cells were cultured in DMEM/F12 (Corning, Manassas, VA, USA) media supplemented with 2 mM GlutaMax, 1% streptomycin-penicillin, 10 µg/mL insulin, 5 µg/mL transferrin, 100 ng/mL cholera toxin, 25 ng/mL epidermal growth factor, 15 mg/500 mL bovine pituitary extract (BPE), 5% FBS, 0.25 µg/mL amphotericin B, and 5 × 10^−7^ M retinoic acid (48). All the cell lines were cultured at 37°C with 5% CO_2_. Procedures involving the handling of cell lines and infectious agents followed the protocols approved by the University of Minnesota Institutional Biosafety Committee (Protocol ID: 2108-39310H).

### Swine field samples

The swine field samples used in this study were archived nasal swabs collected as part of a previously published research (17). These samples were collected from pigs on the same farm (No. 14) during a 6-month surveillance. The selected farm had documented multi-subtype IAV co-circulation with extensive virus genomic diversity during sampling (17). One pig per litter of about 18 days of age was sampled, and 30 pigs were sampled every month from January 2013 to June 2013 for a total of 180 nasal swabs. After collection, the samples were processed, aliquoted, and stored at −80°C. As part of the prior research (17), whole IAV genomes from nasal swabs of 48 pigs were obtained through direct sequencing by Illumina HiSeq 2500 platform, and these samples were used in this study.

### Plaque assay for influenza purification

Plaque assays were used to purify the individual IAV virions following a previously published approach (49). Briefly, the nasal swabs were serially diluted using IAV growth media, which included DMEM media supplemented with 4% bovine serum albumin (BSA) fraction V 7.5% solution (Gibco, Carlsbad, CA, USA), 1% HEPES, 1% streptomycin-penicillin, 50 µg/mL Gentamicin, 50 µg/mL neomycin, and 1.5 µg/mL TPCK-trypsin (Sigma-Aldrich, St. Louis, MO, USA). After washing the MDCK monolayers in 6-well plates using the Hanks’ balanced salt solution (HBSS, BioWhittaker, Verviers, Belgium) that contained 1.5 µg/mL TPCK-trypsin, we inoculated the diluted swab samples on MDCK cells and incubated them for 1 hour in a 5% CO_2_ incubator at 37°C. A 4% agarose gel (Gibco, Carlsbad, CA, USA) was melted in a 70°C water bath and then 1:3 mixed by pre-warmed IAV growth media to make the overlay liquid gel. The overlay liquid gel was kept at 37°C water bath and loaded on the MDCK monolayers when the sample inoculum was removed entirely after the 1-hour incubation. The plates were incubated in a 37°C 5% CO_2_ incubator invertedly when the gel became hardened and waited for the IAV plaques to become visualized in 3 to 5 days. Up to 16 plaques were randomly picked up from each sample using micropipette tips and were stored at −80°C after one passage propagation on MDCK cells.

### Whole-genome next-generation influenza sequencing

The viral RNA of influenza plaques was extracted by using the MagMax viral RNA isolation kit (Ambion, Life Technologies, USA). We performed the conventional one-step reverse transcription-PCR (RT-PCR) amplification on extracted RNA to synthesize the viral cDNA by using the Superscript III One-Step RT-PCR kit with Platinum Taq Polymerase (Invitrogen, Life Technologies, USA) and IAV universal primers (10 µM MBTuni-12 and MBTuni-13) (50). The RT-PCR products were visually inspected by gel electrophoresis and were purified using Qiagen QIAquick PCR purification kit (Qiagen, USA). The eligible viral cDNA products were submitted to the University of Minnesota Genomics Center (UMGC) for whole-genome sequencing. UMGC personnel performed Nanodrop and PicoGreen DNA quantification on the submitted cDNA samples and created the NexteraXT library preparation using the Nextera DNA XT sample preparation kit (Illumina, San Diego, CA, USA). All samples were barcoded and sequenced multiplexed using Illumina NextSeq 2000 P1 flow cell on 2 × 150 bp pair-end mode (Illumina, San Diego, CA, USA).

### Genotype identification of influenza plaques

The raw paired-end reads of the NextSeq sequences that were released from the UMGC were quality checked by FastQC (https://www.bioinformatics.babraham.ac.uk/projects/fastqc/), and the raw reads whose average Q-score per base below 20 in the four base wide of the sliding window were trimmed using Trimmomatic (51, 52). After trimming, we discarded the sequence reads if their lengths were shorter than 36 bp (http://www.usadellab.org/cms/?page=trimmomatic). The trimmed reads were *de novo* assembled by Shovill (https://github.com/tseemann/shovill) (53). The assembled consensus sequences from the plaques were sorted by IAV gene segment, and the MUSCLE program in Geneious Prime software (https://www.geneious.com) was used to align the assembled sequences with the reference sequences (Version commits on 14 May 2021) downloaded from octoFLU (54, 55). The maximum likelihood trees were constructed for each gene segment by IQ-tree and ModelFinder with 1,000 ultrafast bootstraps and best-fitted nucleotide substitution models (56–58). For the virus plaques, we defined the clades of each gene segment based on their branch distance and bootstrap values. The clades were assigned by PhyloPart for each tree if any sub-tree’s median pairwise patristic distance is below a certain percentile threshold of the distribution of the whole-tree patristic distance (59). In addition, the selected clades generally had at least 95% ultrafast bootstrap support values and contained at least two sequences per clade. Occasionally, clades were manually merged to include unassigned sample sequences based on the shared nodes and tree topology. The final partitioned tree clades for each IAV gene segment fell in the following median branch length distance threshold ranges: PB2 – 15.5%–23.5%, PB1 – 17%–23.5%, PA – 17%–20%, H1 – 1.5%–50%, H3 – 10%–16.5%, NP – 18%–18.5%, N1 – 6.5%–48.5%, N2 – 12.5%–18%, M – 22%–48.5%, and NS – 24%–38.5%. Any number within the range set for the median branch length distance threshold for the phylogenetic tree of the corresponding gene segment was assigned the same partitioned tree clade for plaque isolates. The plaques were classified in genotypes according to the assignments of clades on each of their eight gene segments. The genotypic diversity of IAV in single pigs was measured by the Shannon-Wiener index based on the number and the frequency of genotypes identified in a sample (60, 61).

### Differentiation of primary epithelial cells

The sTEC-ALI and NHBE-ALI cells were seeded onto 24-well transwell inserts coated with rat-tail collagen type I (Corning, Bedford, MA, USA). The cells were propagated in their corresponding growth media, which loaded in both apical and basolateral compartments. When the sTEC-ALI and NHBE-ALI cells were grown to 100% confluence in their growth media, we removed the apical medium to expose the sTEC-ALI and NHBE-ALI cells to the air directly. Meanwhile, we replaced the growth media in the basolateral compartment with B-ALI differentiation medium (Lonza, Walkersville, MD, USA) for NHBE-ALI cells and for sTEC-ALI cells, we changed the media to DMEM/F12 supplemented with 2 mM GlutaMax, 2% Nu-Serum (Corning, Bedford, MA, USA), 5 × 10^−7^ M retinoic acid, 1% penicillin-streptomycin, and 0.25 µg/mL amphotericin B (48). After creating the air–liquid interface, the sTEC-ALI and NHBE-ALI cells were kept cultured for 3 to 4 weeks until they fully differentiated at 37°C and 5% CO_2_, with the media changed every 48 hours.

### Influenza growth kinetics on respiratory epithelial cells

The sNEC, NSBE, HNEpC, NHBE, and A549 cells were seeded on 6-well plates as monolayers. The cell monolayers were washed three times using phosphate-buffered saline (PBS) supplemented with 1 µg/mL TPCK-trypsin and then inoculated with the viral isolates at a multiplicity of infection of 0.01 TCID_50_/cell. The inoculum was removed from the plates after a 1-hour incubation at the CO_2_ incubator. The cell monolayers were washed three times using PBS with 1 µg/mL TPCK-trypsin and were cultured in 3 mL MEM (Corning, Manassas, VA, USA) medium supplemented with 1 µg/mL TPCK-trypsin, 1% penicillin-streptomycin, 2 mM GlutaMax, and 1% BSA fraction V 7.5% solution in each well. The cell monolayers were inoculated and cultured at different temperatures to simulate temperatures of various locations in swine and human respiratory tracts (sNEC and HNEpC were cultured at 33°C; NHBE and NSBE were cultured at 37°C; and A549 was cultured at 39°C). We sampled 150 µL supernatants from each well at 1, 24, 48, and 72 hpi and replaced it with the same volume of fresh culture medium.

To test the IAV growth kinetics on epithelial cells at the air–liquid interface, we washed the fully differentiated sTEC-ALI and NHBE-ALI cells in the 24-well transwell inserts for three times using PBS without TPCK-trypsin and inoculated the selected viral isolates diluted in MEM (Corning, Manassas, VA, USA) medium supplemented with 1% penicillin-streptomycin, 2 mM GlutaMax, and 1% BSA fraction V 7.5% solution on the apical surface at a multiplicity of infection of 0.1 TCID_50_/cell. After a 2-hour incubation at 37°C in a CO_2_ incubator, we removed the inoculum and washed the apical surface for three times with PBS without TPCK-trypsin. The sTEC-ALI and NHBE-ALI cells were cultured at 37°C and 5% CO_2_ for the indicated sampling times. To collect the virus samples from the differentiated cells, we added 150 µL MEM (Corning, Manassas, VA, USA) medium supplemented with 1% penicillin-streptomycin, 2 mM GlutaMax, and 1% BSA fraction V 7.5% solution to the apical cell surface and incubated at 37°C and 5% CO_2_. The apical supernatants were harvested after a 30-minute incubation (48). For the sTEC-ALI and NHBE-ALI cells in each 24-well transwell inserts, the supernatants were collected at 3, 24, 48, and 72 hours postinoculation.

All the supernatants were titrated on MDCK cells by TCID_50_/mL and titers calculated by the Reed and Muench method (62). The growth kinetics of any selected IAV plaque were evaluated in triplicate.

### Statistical analysis

To compare the overall virus growth ability between different IAV plaques on certain cells, we calculated the AUC for the viral load (log_10_ transformed) of the plaques for each replicate from 1 (for virus testing at cell monolayers) or 3 hpi (for virus testing at air–liquid interface) to 72 hpi. We set the virus titer at the first sampling time as baseline to compute the AUC to exclude the background virus left from the inoculation. Statistical significance of the AUC between the plaques and cell lines was assessed by two-way analysis of variance (ANOVA) with Tukey Honestly Significant Difference (HSD) adjustment for multiple comparisons. Statistical analyses were performed separately on the AUC data generated from the IAV growth curves on the cells at the monolayer and air–liquid interface conditions. Results were considered significant at *P* < 0.05.

## Data Availability

All the raw sequence reads of influenza viruses generated in this study have been deposited in Sequence Read Archive (SRA) of National Center for Biotechnology Information (NCBI) under BioProject accession numbers PRJNA1106994.

## SUPPLEMENTAL MATERIAL

Fig. S1 mbio.01924-24-s0001.pdf Phylogenetic analysis and clade assignment for influenza gene segments from isolated plaques.

ASM does not own the copyrights to Supplemental Material that may be linked to, or accessed through, an article. The authors have granted ASM a non-exclusive, world-wide license to publish the Supplemental Material files. Please contact the corresponding author directly for reuse.
