# Supplementary material for: Naturally occurring influenza reassortment in pigs facilitates the emergence of intrahost virus subpopulations with distinct genotypes and replicative fitness
Source: mBio. 2024 Nov 29;16(1):e01924-24. doi: 10.1128/mbio.01924-24 (PMC11708028; doi:10.1128/mbio.01924-24)
Supplement: Fig. S1 — Phylogenetic analysis and clade assignment for influenza gene segments from isolated plaques. [file mbio.01924-24-s0001.pdf]

Supplemental Material

PB2

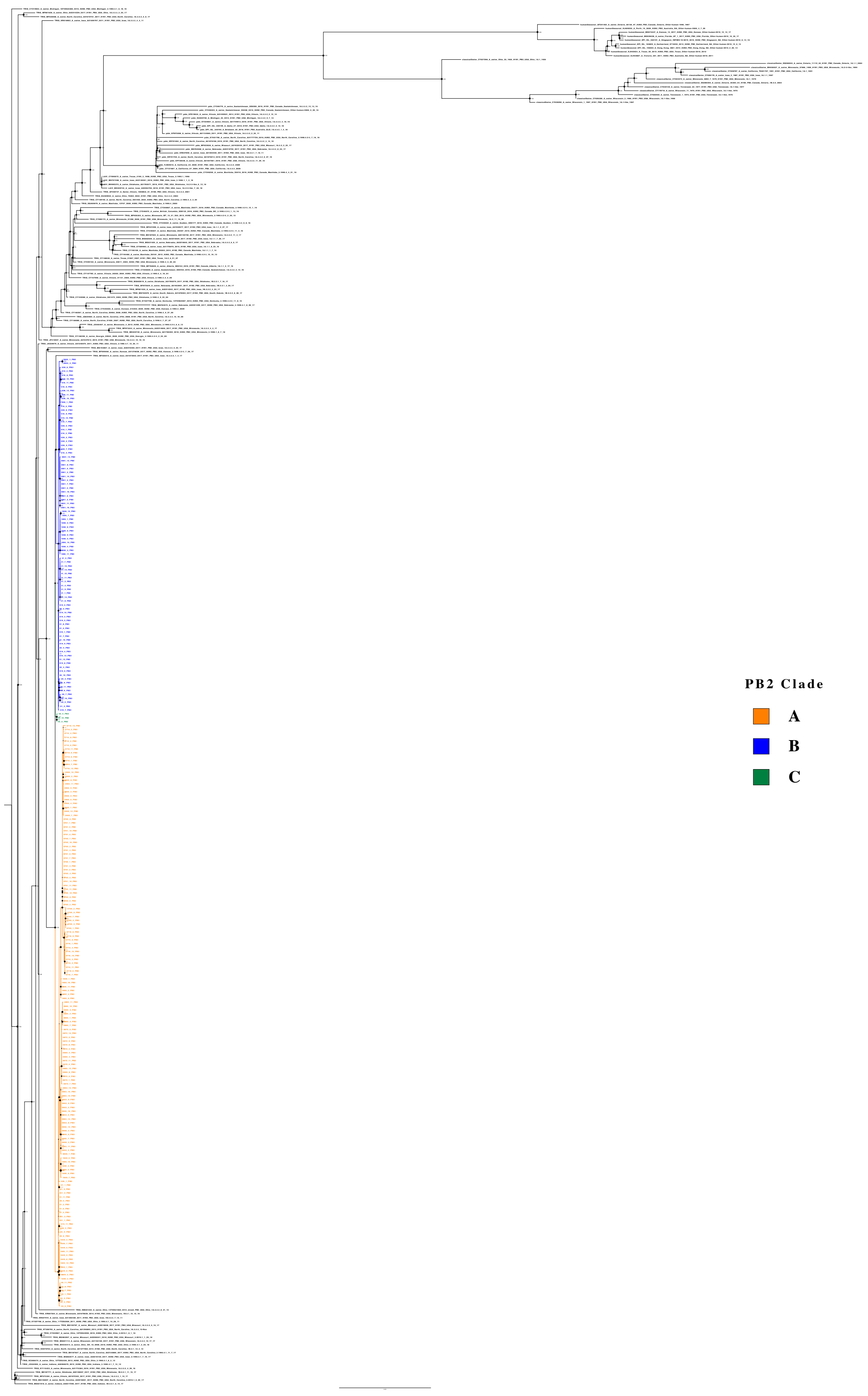

PB1

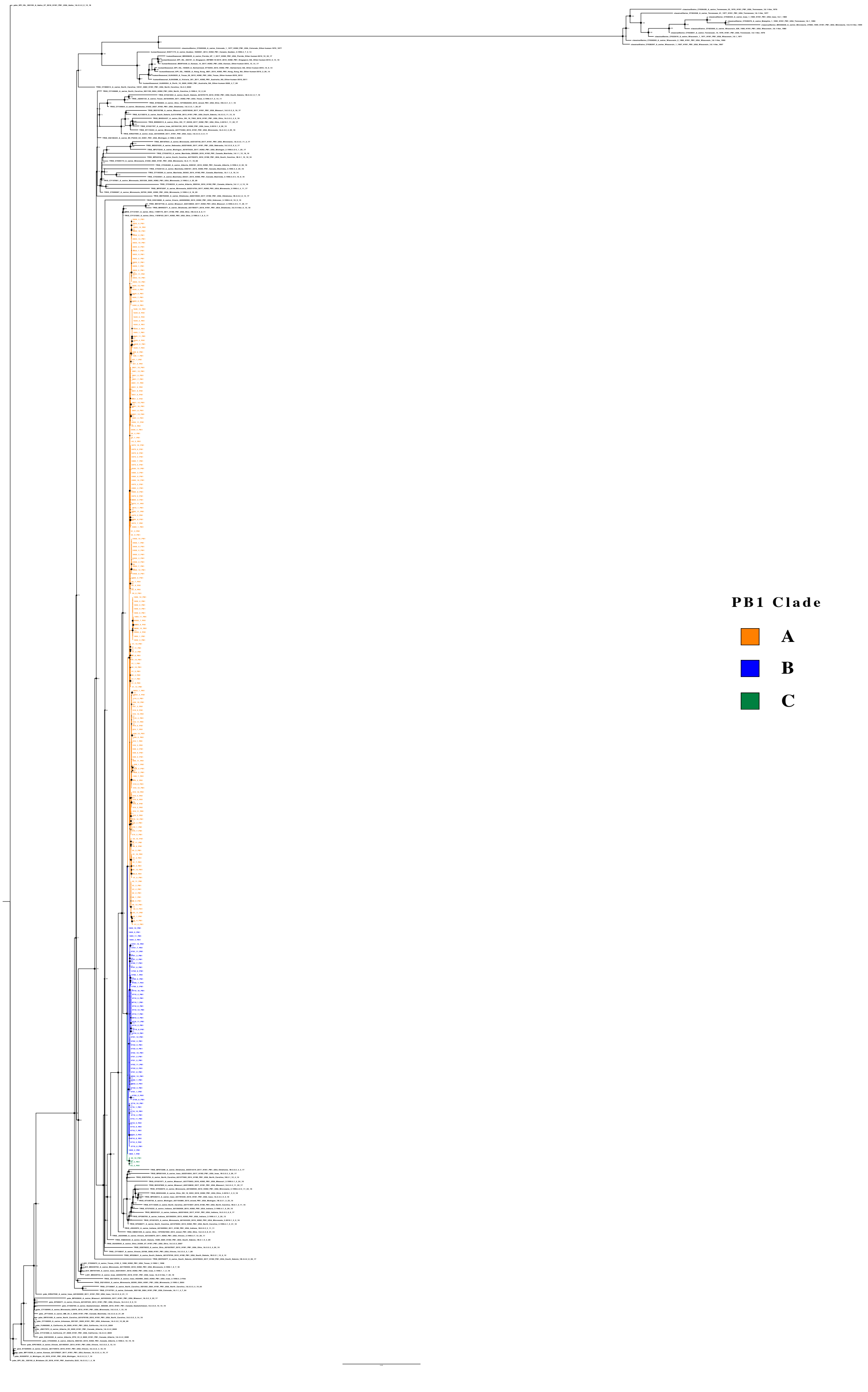

PA

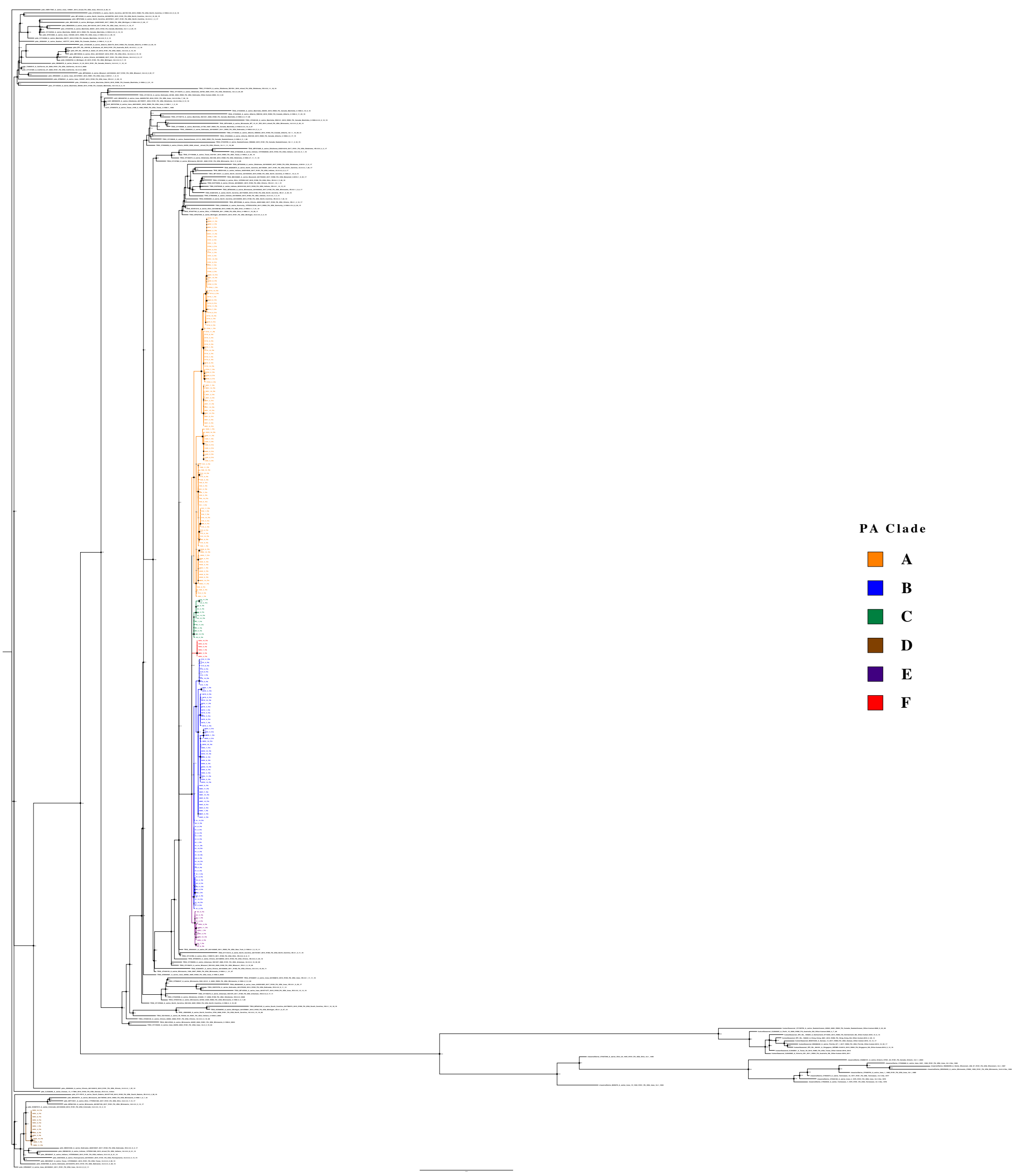

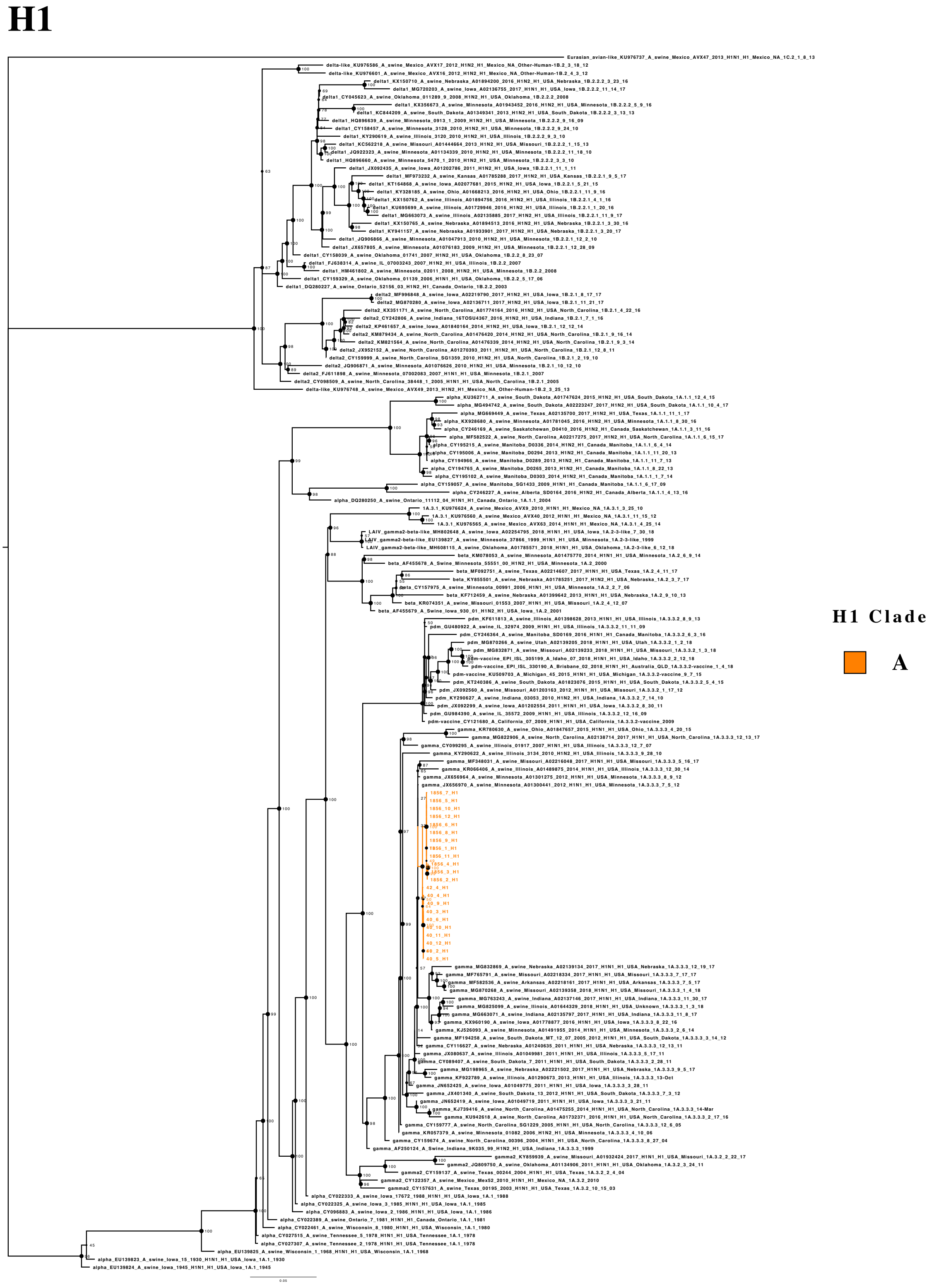
H1
H1 Clade
A

H3

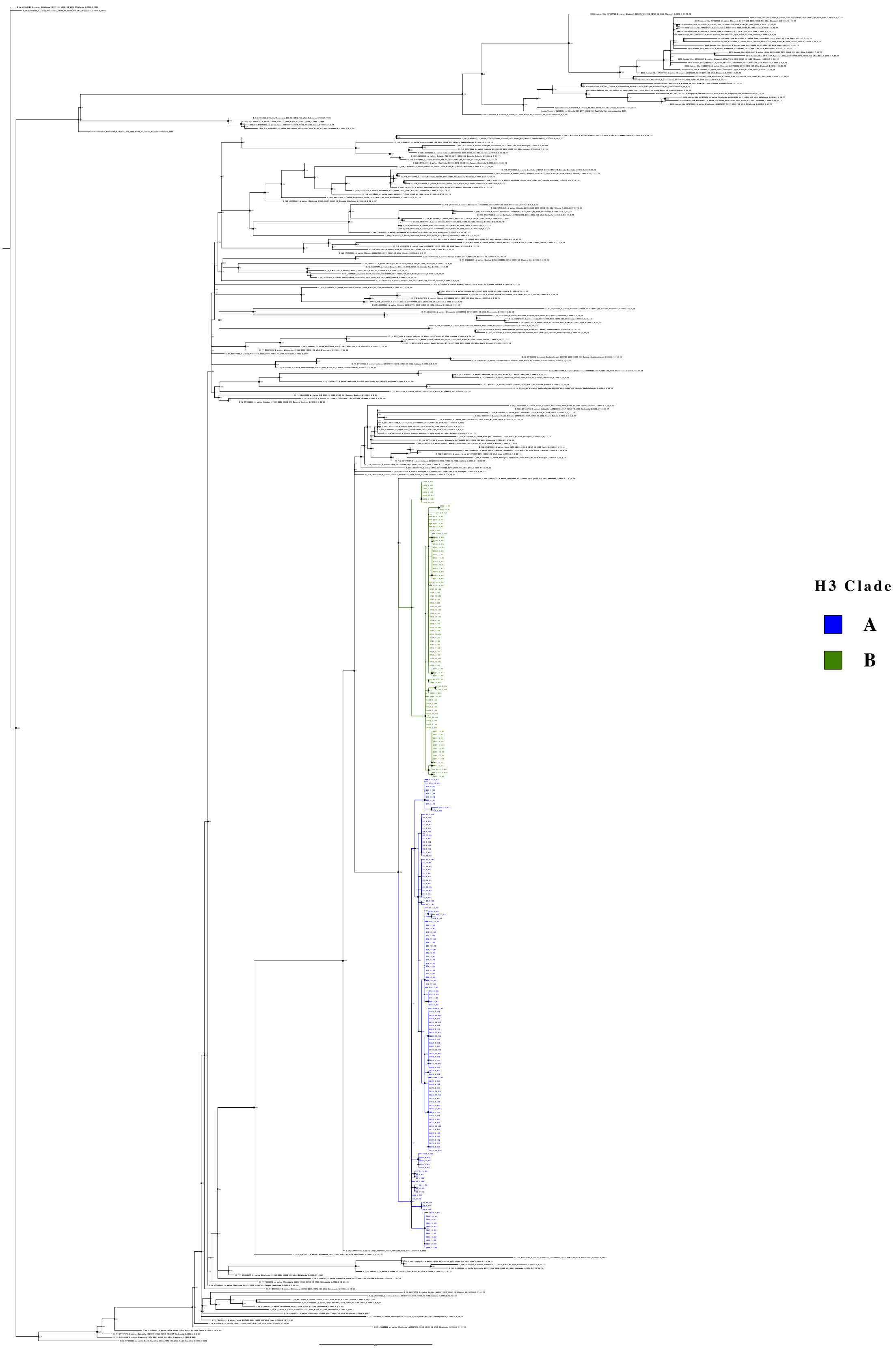

NP

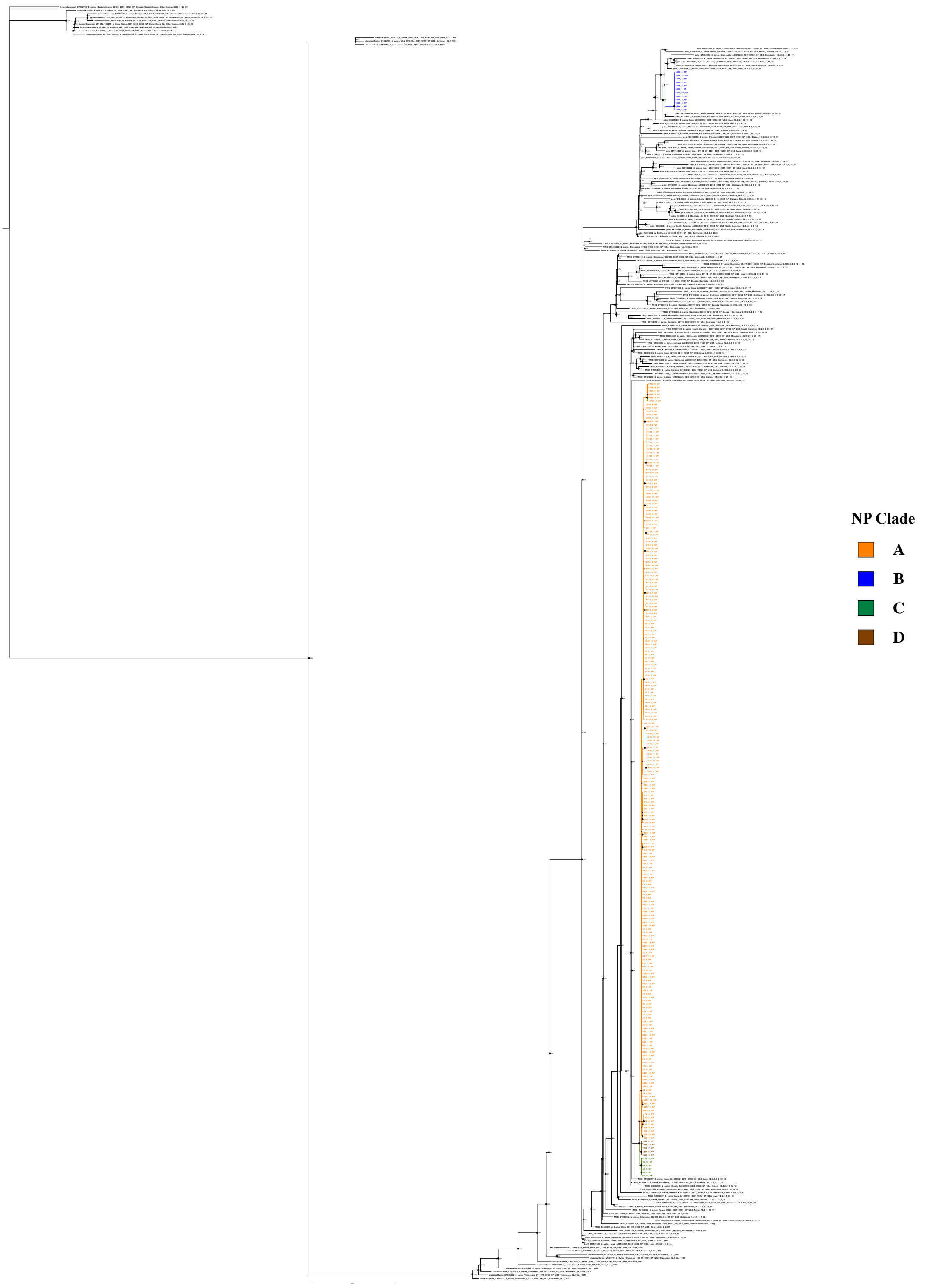

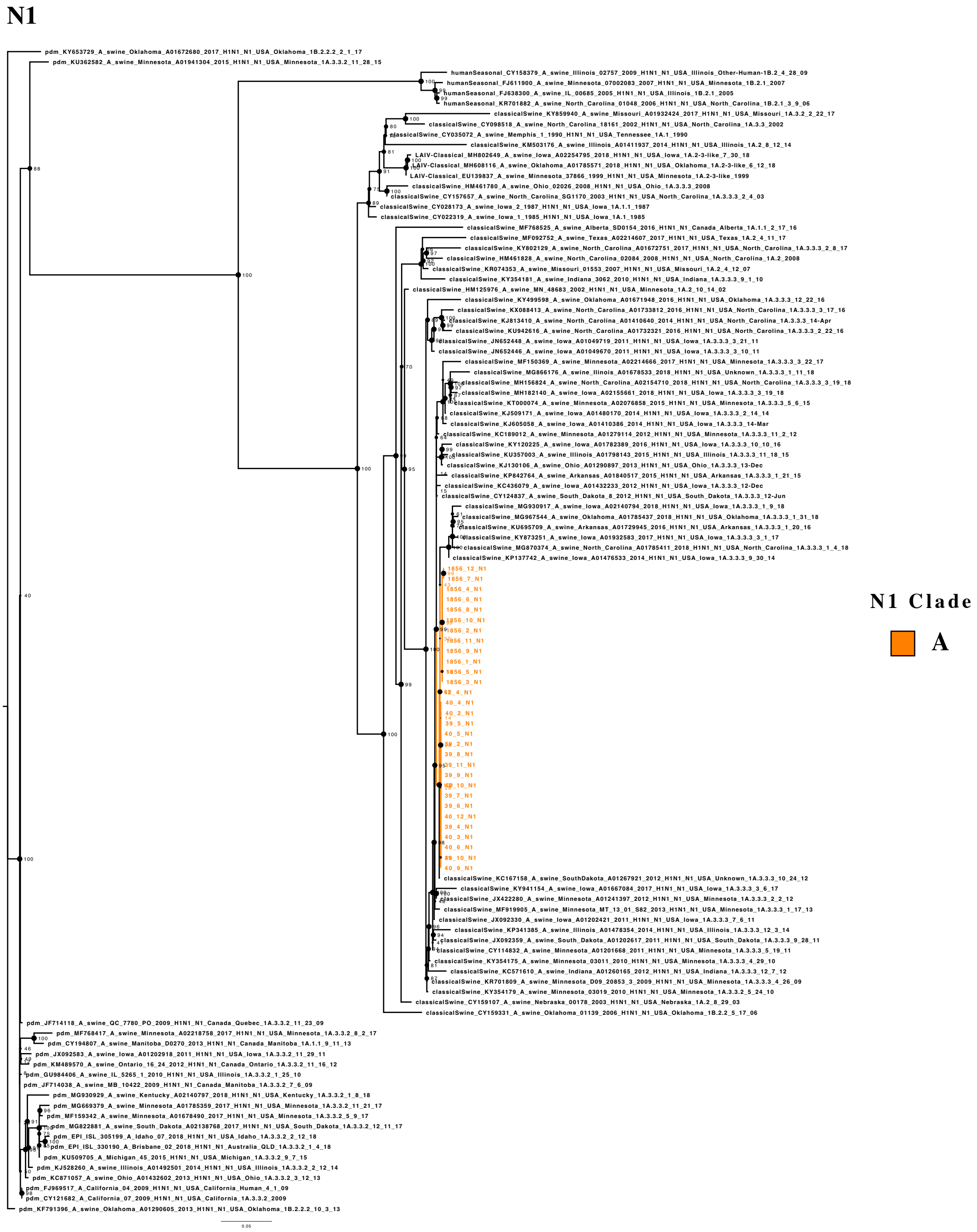
N1
N1 Clade
A
0.05

N2

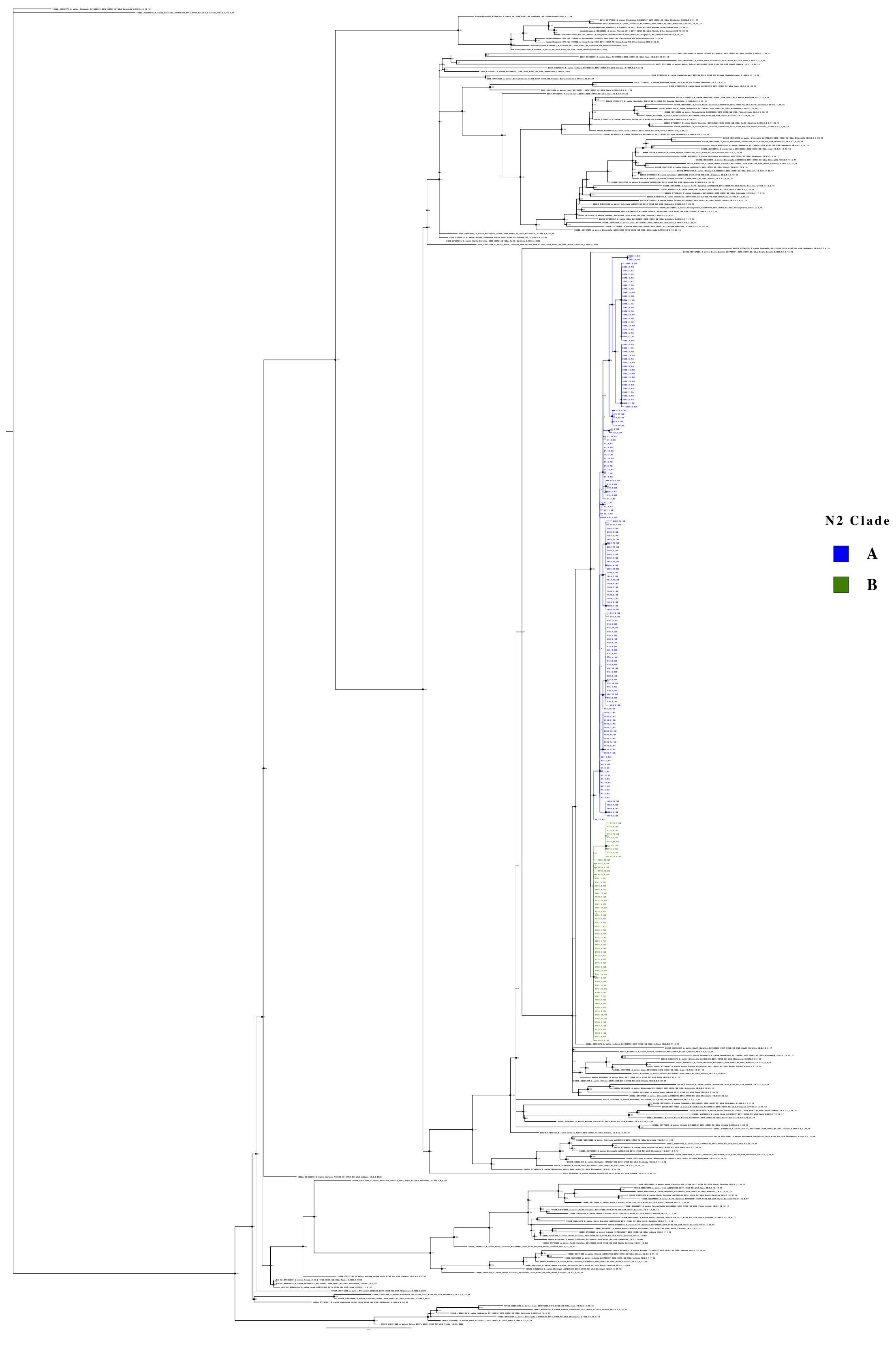

M

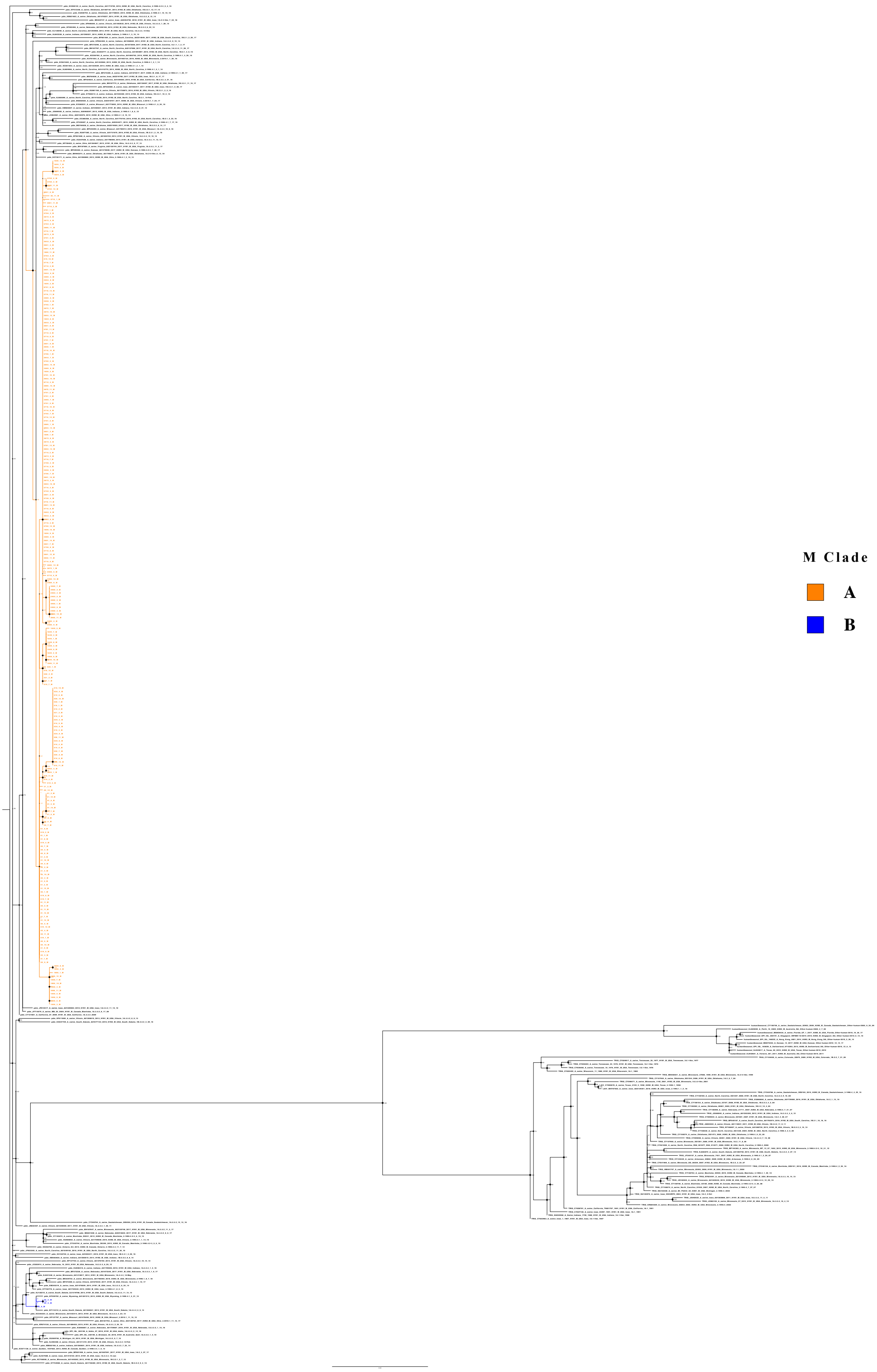

NS

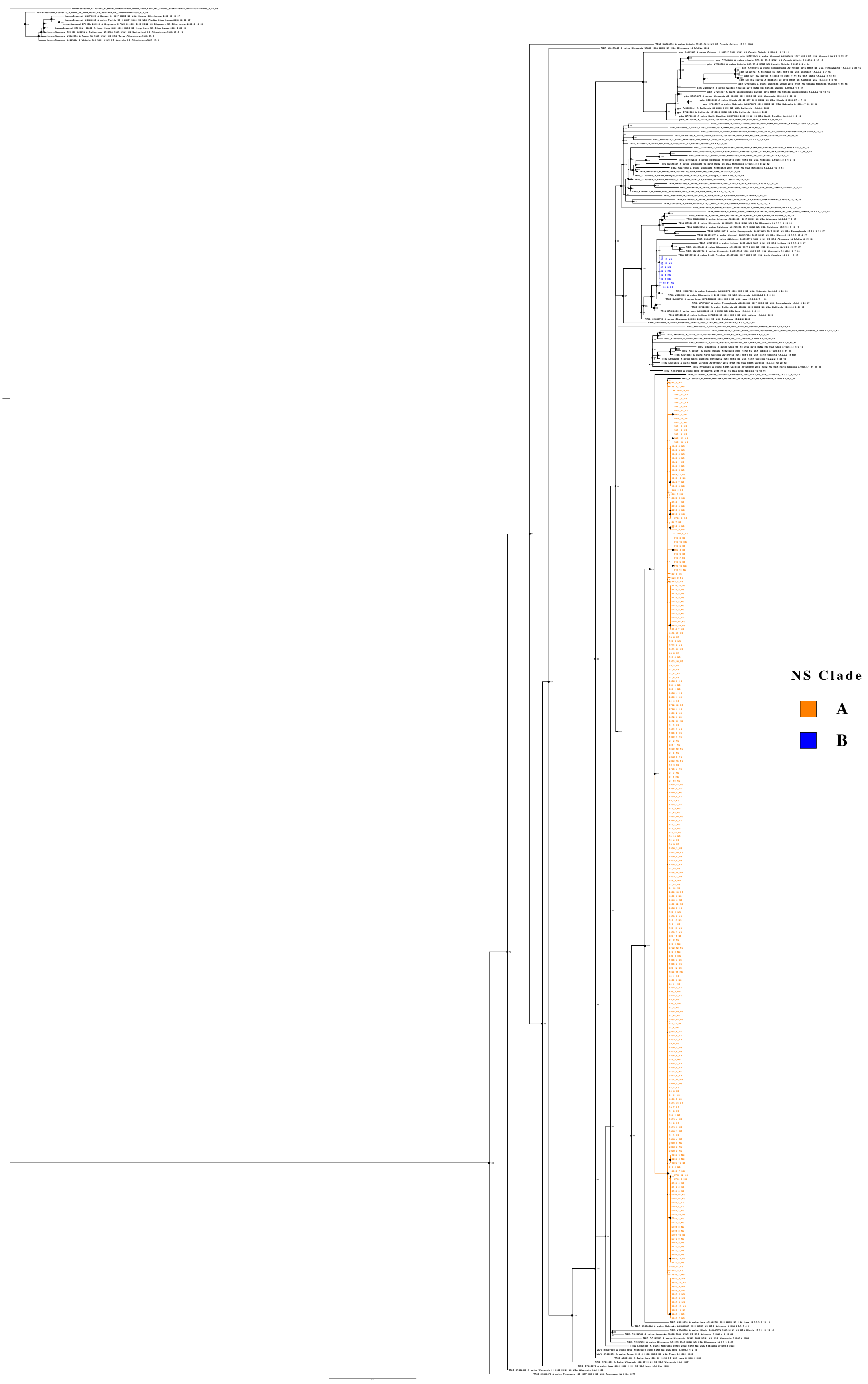

Fig. S1: Phylogenetic analysis and clade assignment for influenza gene segments from isolated plaques. Trees were constructed by the maximum likelihood method using the assembled sequences from isolated influenza plaques and the reference sequences from octoFLU. The colors of branches and taxa indicate the specific clades assigned for the influenza gene sequences from isolated plaques. The bootstrap values on the tree nodes are showed and represented proportionally by the size of the circles.
